# Perineural Dexmedetomidine as an Adjuvant Reduces the Median Effective Concentration of Lidocaine for Obturator Nerve Blocking: A Double-Blinded Randomized Controlled Trial

**DOI:** 10.1371/journal.pone.0158226

**Published:** 2016-06-24

**Authors:** Yuechun Lu, Jian Sun, Xinqi Zhuang, Guoyi Lv, Yize Li, Haiyun Wang, Guolin Wang

**Affiliations:** 1 Department of Anesthesiology, Second Hospital of Tianjin Medical University, Tianjin, China; 2 Department of Anesthesiology, Tianjin Medical University General Hospital, Tianjin, China; Eberhard Karls University, GERMANY

## Abstract

Research suggests that the addition of dexmedetomidine to local anesthetics can prolong peripheral nerve blocks; however, it is not known whether dexmedetomidine can reduce the quantity of local anesthetic needed. We hypothesized that adding dexmedetomidine as an adjuvant to an obturator nerve block could reduce the median effective concentration of lidocaine. In this double-blinded randomized trial, 60 patients scheduled for elective transurethral resection of bladder tumors on the lateral wall were randomly divided into two groups: the control group (C group, n = 30) and the dexmedetomidine group (D group, n = 30). Two main branches of the obturator nerve (i.e., anterior and posterior) were identified using neural stimulation at the inguinal level, with only lidocaine used for the C group and 1 μg/kg dexmedetomidine combined with lidocaine used for the D group. The median effective concentration was determined by an up-and-down sequential trial. The ratio of two consecutive concentrations was 1.2. The median effective concentration (95% confidence interval) of lidocaine was 0.57% (0.54%-0.62%) in the C group and 0.29% (0.28%-0.38%) in the D group. The median effective concentration of lidocaine was significantly lower in the D group than in the C group (p < 0.05). These results indicate that dexmedetomidine (1 μg/kg) in combination with lidocaine for obturator nerve block decreases the median effective concentration of lidocaine.

***Trial Registration*:** ClinicalTrials.gov NCT02066727

## Introduction

Transurethral resection of bladder tumors (TUR-BT) involving the lateral wall, performed with a resectoscope, entails the risk of stimulating the obturator nerve [1]. The nerve passes close to the lateral bladder wall and its stimulation results in a sudden, violent thigh adductor spasm. The complications of thigh adductor spasm, such as bladder perforation, dissemination of cancer cells [2], and vascular injury [3], are disastrous and even life-threatening. Different strategies have been adopted to avoid these complications during surgery, such as use of general anesthesia with muscle relaxants [4] or the use of obturator nerve block (ONB) [5, 6]. Selective ONB along with intervertebral anesthesia is effective for preventing thigh adductor spasm [5, 6] as well as to avoid the complications of general anesthesia in older patients. Because a local anesthetic is needed in both intervertebral block and ONB, the quantity of local anesthetic should be considered to avoid intoxication, especially for patients who need an ONB on both sides. In general, it is better to use the least amount of anesthesia possible.

Recently, dexmedetomidine has been found to be an effective adjuvant to local anesthetics in peripheral nerve blocks. While most previous research focused on regional techniques in the upper limbs [7–10], reports of its use in the lower limbs are limited [11], and its use in obturator plexus block has not been reported. The addition of dexmedetomidine to nerve plexus blocks resulted in a shorter onset time [7–9], an extended duration [7–9, 11], and a longer postoperative pain-free period [7–9, 11]. It is uncertain whether dexmedetomidine could decrease the quantity of local anesthetic required. In this study, we hypothesize that dexmedetomidine could reduce the quantity of local anesthetics in peripheral nerve blocks. The aim of the present study was to investigate the effects of dexmedetomidineas anadjuvant on the median effective concentration (EC50) of lidocaine for ONB during transurethral resection of bladder tumors.

## Materials and Methods

### Patient Enrollment

In this prospective, double-blinded, single-center randomized controlled trial, 60 patients with American Society of Anesthesiologists (ASA) physical status I and II (53 male and seven female patients) and a mean age of 64.5 years (range, 18–80 years), who were scheduled to undergo elective TUR-BT involving the lateral wall between February 2014 and May 2014 in Second Hospital of Tianjin Medical University (Tianjin, China), were enrolled in the study. We excluded patients with a known allergy to local anesthetics or other medications used in this study. Patients with neuromuscular disease, diabetes mellitus, alcohol or drug abuse, or abnormal coagulation function were also excluded.

### Ethics Statement

This study was approved by the Committee on the Ethics of Experiments of Tianjin Medical University General Hospital (Tianjin, China; Permission Number IRB2013-037-01). Prior written and informed consent was obtained from every participant. Trial registration was performed via the ClinicalTrials.gov registry (ID:NCT02066727).

### Grouping and Trial Protocol

Using a computer-generated sequence of numbers, patients were randomly assigned to the lidocaine group (group C, 30 patients) or the dexmedetomidine group (group D, 30 patients); they were blinded to their group assignment. In group C, only lidocaine was used in ONB, while in group D, a mixture of lidocaine and 1 μg/kg dexmedetomidine was used (S1 Protocol). The randomized sequence numbers were stored in sealed opaque envelopes. If the patient fulfilled the inclusion criteria, the investigator opened the sealed envelope in the operating room. After that, the prepared corresponding drug was provided to the doctor for the ONB.

The Dixon “up-and-down” sequential allocation method [12] was used to determine the EC50 of lidocaine, and the two groups were run in parallel. The concentration of lidocaine for the second and subsequent patients in each group was dictated by the response of the previous patient in the group, such that an effective block led to a decrease by a factor of 1.2 in concentration for the next patient, and an ineffective block led to an increase by a factor of 1.2. A technical failure led to the exclusion of the patient from analysis, and replacement by the next recruited patient, using the same concentration. The initial concentration of lidocaine was 1.5%, and 1:200,000 epinephrine (Jinyao Pharmaceutical Co. Ltd., Tianjin, China) was added to lidocaine in all patients. In the Dixon method, the stopping rule requires at least six failure/success pairs. In this study, seven crossovers were considered sufficient to identify the EC50 of lidocaine. The required failure/success crossovers were achieved with 24 patients in group C and 26 patients in group D. As a result, 30 patients were enrolled in each group.

### Obturator Nerve Block

Patients were administered 0.5 mg atropine (Jinyao Pharmaceutical Co. Ltd., Tianjin, China) intramuscularly before the block. After the insertion of a 20-gauge intravenous cannula (B. Braun Medical Inc., Melsungen, Germany) in the arm, a 5 mL/kg/h infusion of lactated Ringer's solution (Otsuka Pharmaceutical Co. Ltd., Tianjin, China) was started. After standard anesthesia monitoring, baseline measurements of electrocardiogram, noninvasive arterial blood pressure, peripheral oxygen saturation, and respiratory rate were recorded before the block was performed. The ONB was performed on the right or/and left sides, according to the approach assignment for the side. All ONB procedures were performed by a single experienced doctor who did not know the group allocation and was not involved in further perioperative care of the patients.

The point for puncture was identified and ONB was performed, as previously described [13,14]. The patient was placed supine and the leg was slightly abducted. A line was then drawn between the inner border of the adductor longus tendon and the most obvious point of the femoral arterial pulse, along the inguinal crease on the skin. The midpoint was marked for puncture. A 21-gauge 100-mm needle (B. Braun Medical Inc., Melsungen, Germany), which was connected to a peripheral nerve stimulator (B. Braun Medical Inc., Melsungen, Germany), was inserted in a 30° cephalad direction to the skin. Initially, a current of 2 mA at a frequency of 2 Hz was set. Once the needle was in contact with the anterior branch of the obturator nerve, gracilis or adductor longus contraction was elicited. The current was gradually reduced until muscle contractions occurred at 0.4–0.5 mA. Following negative aspiration, 5 mL lidocaine (Jinyao Pharmaceutical Co. Ltd., Tianjin, China), with or without dexmedetomidine, was injected. The needle was then inserted more deeply in a 5° lateral direction, until an adductor magnus contraction was elicited, which indicated the posterior branch was detected. The remainder of the operation was the same for the anterior branch.

### Block Evaluation

#### Leg lift scale

The patients were rated, as follows: 0 = patient lifts his leg without abduction and adducts his leg without effort; 1 = patient lifts his leg with slight abduction and adducts his leg with small effort; 2 = patient lifts his leg with obvious abduction and adducts his leg with much effort; 3 = patient lifts his leg with much effort and obvious abduction and/or cannot adduct his leg at all.

#### Measurement of the abductor muscle strength

After placing a mercury sphygmomanometer (previously inflated to 40 mmHg) between the patient’s knees, the patient was asked to stretch his leg and squeeze the blood pressure cuff with maximum effort by adducting the leg. During the exercise, the observer fixed the patient’s contralateral leg toward the midline to ensure that the pressure generated on the cuff reflected only the strength of the blocked leg. The maximal sustained pressure read on the mercury sphygmomanometer was regarded as the adductor muscle strength of the blocked leg [15].

#### Evaluation of leg movement during the operation

Leg movement was evaluated as follows: 0 = leg abducted violently during resection of the bladder tumor and the operation was difficult to continue; 1 = leg abducted slightly during resection of the bladder tumor and the operation could continue with care; 2 = leg did not move at all during the resection of the bladder tumor.

All aforementioned evaluations were performed by a doctor who was blinded to the group allocation. The identification of a successful ONB should satisfy all the following standards within 10 minutes after ONB: leg lift scale rated “2” or “3”, adductor muscle strength decreased ≥50%, and leg movement evaluation rated “2”. In a failure case, intravenous cisatrocuronium (Hengrui Pharmaceutical Co. Ltd., Jiangsu, China) was administered at 10 mg or more, if necessary, before the operation.

### Anesthetic Management

As compensation for nerve block and to facilitate postoperative evaluation, general anesthesia was scheduled after the block evaluation. Induction was performed and maintained by propofol (Guorui Pharmaceutical Co. Ltd., Sichuan, China) and remifentanil (Renfu Pharmaceutical Co. Ltd., Hubei, China) intravenously. Ventilation was sustained with a laryngeal mask airway (Medis Medical Device CO. Ltd., Tianjin, China). Perioperative monitoring included pulse oximetry, electrocardiogram, noninvasive arterial pressure, bispectral index, temperature, and partial pressure of carbon dioxide in end-expiratory gas. Liquid for bladder flushing was heated to 30°C in order to maintain normal body temperature. If the heart rate declined to less than 40 bpm, or decreased more than 30% of the base value, it was recorded as bradycardia and intravenous atropine was administered.

The laryngeal mask airway was removed postoperatively after the recovery of patient’s spontaneous breathing, consciousness, and physical reflexes. Delayed recovery was defined as when the laryngeal mask airway could not be removed 30 minutes after the operation. The time interval between the completion of ONB and TUR-BT was recorded. In the postanesthesia care unit (PACU), adductor muscle strength was evaluated again after performing a successful block. Within 24 hours after the study, patients were examined to identify adverse events such as persistent groin pain, painful paresthesia, and neuropathy.

### Statistical Analysis

In addition to the goal of estimating the EC50 of lidocaine from the up-and-down study, it is also necessary to specify the precision of the target dose with a 95% confidence interval (CI). The drug effect increases with increasing concentration, but biological and experimental variability may produce unexpected changes in the observed response rate as concentration increases. Therefore, the adjusted response rates were calculated by the pooled-adjacent-violators algorithm (PAVA). The isotonic estimator of target dose with isotonic regression provides a smaller bias, mean square error, and greater precision (i.e., tighter CI), and requires no symmetry assumption [16]. The EC50 of lidocaine and CIs were calculated from R version 3.0.1 software (R Foundation for Statistical Computing, Vienna, Austria). Comparison of EC50 between groups used the method of isotonic regression.

For all patients, sex, age, body mass index, ASA grade, time interval between the completion of ONB and TUR-BT, adductor muscle strength evaluation after ONB and in the PACU, and the occurrence of bradycardia, delayed recovery, groin pain, painful paresthesia, and neuropathy, were recorded. The data are presented as the mean ± the standard deviation, or as the count, as appropriate. Statistical comparisons of continuous variables were performed using Student’s t-test, and categorical variables were analyzed with the χ^2^ test using SPSS software, version 19.0 (SPSS, Inc., Chicago, Illinois, USA). Significance was defined at p < 0.05.

## Results

### Characteristics of the Patients and Adverse Effects

**Fig 1:**
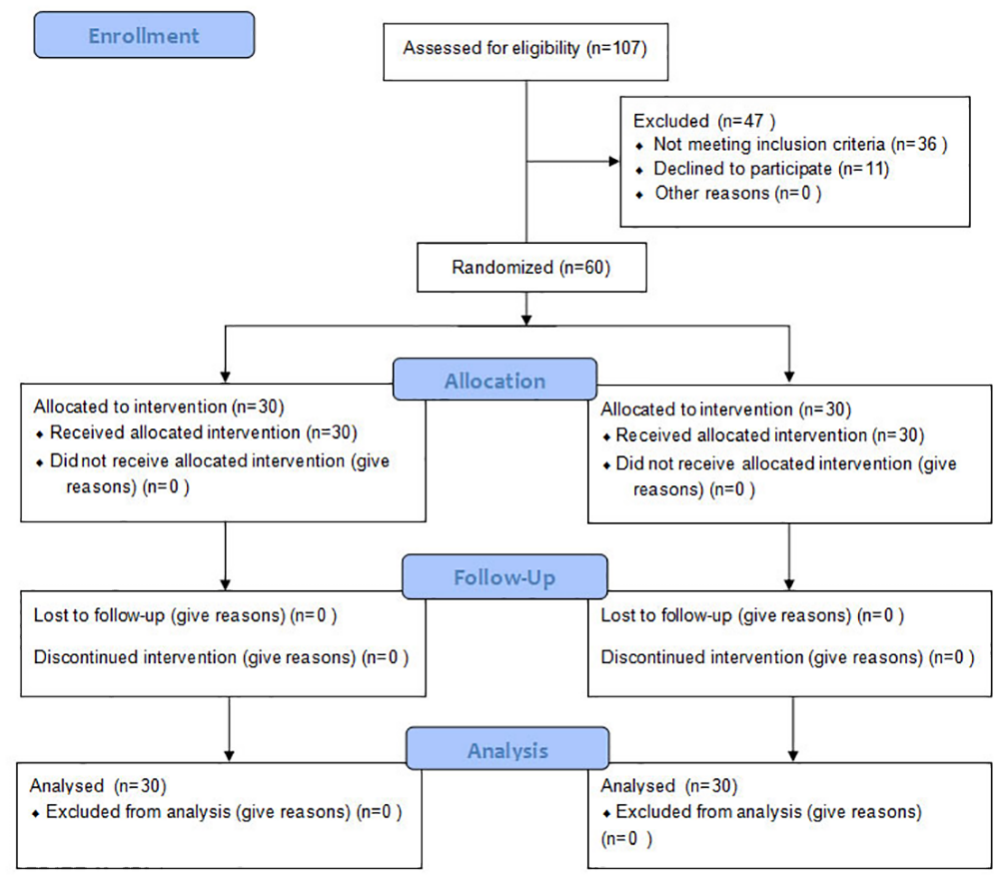
Trial flow chart.

The trial profile is shown in the trial flow chart (Fig 1). Sixty patients were enrolled and completed the study. The groups were similar with regard to sex, age, body mass index, ASA grade, and time interval between the completion of ONB and TUR-BT (p > 0.05), as shown in Table 1. Adductor muscle strength evaluation was similar between groups after ONB and in the PACU (p > 0.05), as shown in Table 2. No bradycardia or delayed recovery occurred in either group 30 minutes after the operation, and no persistent groin pain, painful paresthesia, or neuropathy was detected 24 hours postoperatively.

**Table 1:** Patient Variables.

Variables	C group (n = 30)	D group (n = 30)	p value
Age, years, mean ± SD	63.2 ± 12.1	65.9 ± 10.4	0.371
BMI, kg/m^2^, mean ± SD	25.4 ± 2.8	24.5 ± 3.3	0.252
Gender, F/M	27/3	26/4	0.688
ASA grade, I/II	16/14	14/16	0.606
Time interval between completion of ONB and TUR-BT, minutes, mean ± SD	54.5 ± 32.5	61.8 ± 25.7	0.336

*Note*. ASA = American Society of Anesthesiologists, BMI = body mass index, F = female, M = male, ONB = obturator nerve block, TUR-BT = transurethral resection of bladder tumors.

**Table 2:** Adductor Muscle Strength Evaluation.

Muscle Strength Evaluation	C group (n = 30)	D group (n = 30)	p value
After ONB, mmHg, mean ± SD	57.7 ± 15.4	59.0 ± 13.4	0.721
In PACU, mmHg, mean ± SD	70.2 ± 23.4	67.8 ± 15.5	0.650

*Note*. ONB = obturator nerve block, PACU = postanesthesia care unit, SD = standard deviation.

### Difference in EC50

**Fig 2:**
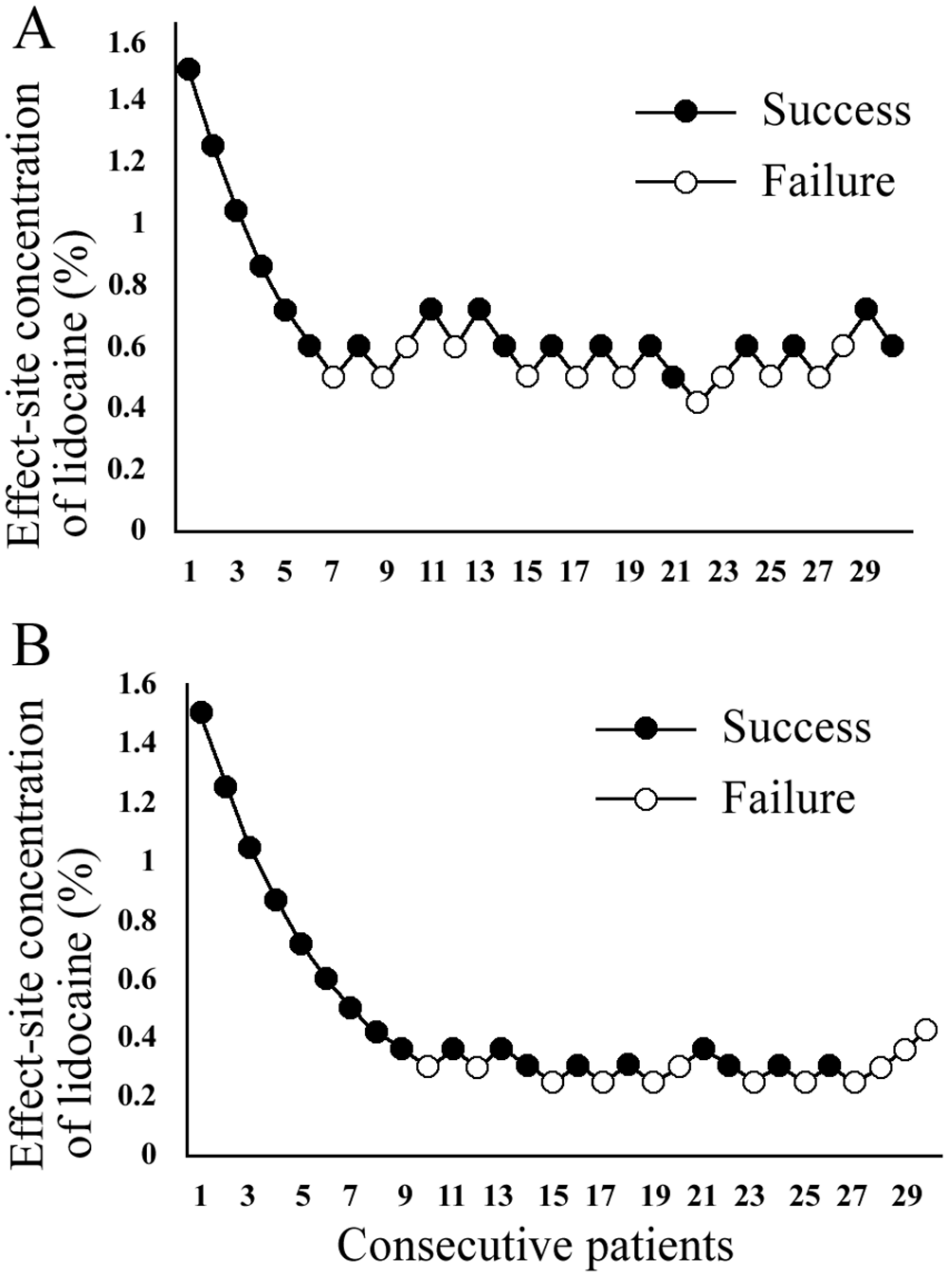
Dose-response data of consecutive patients for both groups. The sequences of positive responses (solid dots) and negative responses (hollow dots) recorded in consecutive patients (A) for the C group and (B) for the D group.

**Fig 3:**
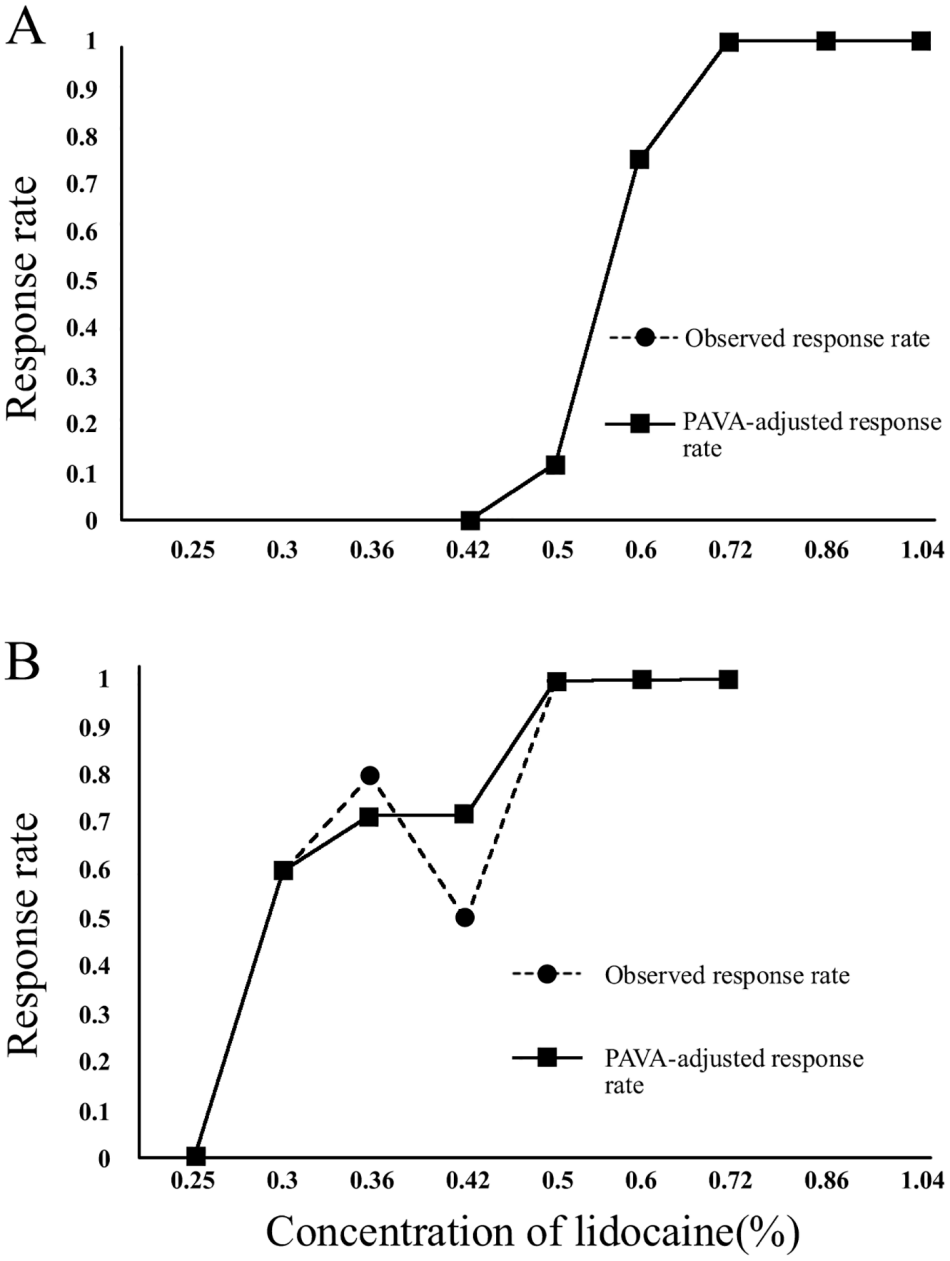
Observed and pooled-adjacent-violators algorithm response rate. The estimator of the observed response rate (round dots, dotted line) and the PAVA-adjusted response rate (squares, solid line) at various lidocaine concentrations are plotted on the y-axis (A) for the C group and (B) for the D group. PAVA = pooled-adjacent-violators algorithm.

The sequences of positive and negative responses recorded in consecutive patients for both groups are shown in Fig 2A and 2B. To provide better precision and narrow CIs for the point estimates, isotonic regression estimation and the PAVA approach were used. Nerve block failed when lidocaine was decreased to 0.50% in the C group and 0.29% in the D group, which were used as the first case for calculation. The EC50 of lidocaine required for ONB was 0.57% (95% CI, 0.54%-0.62%) in the C group, which is higher than that for D group [0.29% (95% CI, 0.28%-0.38%); p < 0.001] (Fig 3A and 3B).

## Discussion

Dexmedetomidine is increasingly used as a local anesthetic to improve the block quality in peripheral nerve blocks. Brummett reported that a large dose of dexmedetomidine enhances the duration of bupivacaine for the sciatic nerve block in rats [17]. In other studies, he reported that dexmedetomidine could also enhance blockage when added to ropivacaine in a dose-dependent fashion [18], most likely because of the blockage of hyperpolarization-activated cation current (Ih current) [19,20]. Human studies were performed in addition to animal studies. The use of dexmedetomidine provided faster onset and longer duration when added to local anesthetic for brachial plexus block [7,9,10], ulnar nerve block [8], posterior tibial nerve sensory blockade [11], and caudal block [21]. In this study, we found that dexmedetomidine could also be used as an adjuvant for ONB; moreover, dexmedetomidine could reduce the quantity of local anesthetic needed.

A dosage of 1 μg/kg dexmedetomidine was chosen for ONB, based on data from previous studies in which it was used peripherally [10,11] or caudally [21]. This dose has been proven safe intravenously [22], and an animal study showed that even high doses of dexmedetomidine did not damage axons or myelin sheaths, and could even reduce bupivacaine-induced acute perineural inflammation [17].

Different approaches have evolved for ONB. The classic approach has been used for years, but it is quite invasive, technically difficult, and requires a longer needle. The direction of the needle is toward the pelvic contents, which increases the chance of complications. In 2005, Choquet described the inguinal approach [23]. This approach, which provides adequate blockage like the classic approach, was reported to be easier [13] and safer [14] than the classic approach. We used the inguinal approach in this study, which resulted in successful nerve block with no complications detected.

Because the cutaneous distribution of the obturator nerve is variable, the evaluation of effective ONB can only be based on the function of adductor muscles [24]. A successful ONB should lead to obvious abduction in the leg, but the rectus femoris can partially perform the abduct function in the lower limb. Because we observed that leg lift scale ratings were typically between 1 and 2 for a few patients, the leg lift scale alone could not be the only evaluation for an ONB. In some studies, successful ONB was defined as no leg movement during TUR-BT [25]. However, patients who receive insufficient ONB may not exhibit any obturator nerve reflex because the resection may be located far from the lateral wall. For these reasons, we used the leg lift scale, adductor muscle strength measurement, and leg movement evaluation as multiple methods to evaluate the efficacy of ONB, in order to ensure accuracy.

A number of previous studies have been performed to investigate dexmedetomidine as an adjuvant for nerve blocks. Local vasoconstriction caused by α-2-adrenoceptor agonists prolonged the duration of local anesthetic and improved the quality of analgesia [26, 27]. Brummett showed that the analgesic effect of peripheral dexmedetomidine might be caused by inhibition of the hyperpolarization-activated cation current (I_h_), which prevented the nerve returning from a hyperpolarized state to resting membrane potential for subsequent firing [20]. Blocking the I_h_ current will result in prolonged hyperpolarization of the nerve, which seems to be more distinct in the unmyelinated C (i.e., pain) fibers than in α (i.e., motor) fibers. Furthermore, Maruta found that dexmedetomidine can inhibit the function of Na(v)1.7, independent of α-2-adrenoceptor in adrenal chromaffin cells [28]. This effect could not be prevented by a perfused α-2-receptor antagonist, which may imply the mechanism of the peripheral antinociceptive effects of α-2-agonists [28]. Chen discovered that dexmedetomidine is not limited to its interactions with α-2-adrenoceptors [29]. Inhibitory effects on delayed rectifier K^+^ current I(K(DR)) and Na^+^ current I(Na) constitute one of the underlying mechanisms through which dexmedetomidine might affect neuronal activity. The mechanism by which dexmedetomidine reduces the quantity of lidocaine needed for ONB may lie in the aforementioned reasons; however, this study did not address the mechanisms underlying the observed effects.

Dexmedetomidine can cause dose-dependent side effects such as bradycardia and hypotension [30,31]. We found that 1 μg/kg perineural dexmedetomidine did not cause hemodynamic adverse effects. In studies using 1 μg/kg dexmedetomidine as an adjuvant, Song discovered that heart rate was significantly lower at 40 minutes after the drug injection [10], and Rancourt observed that blood pressure decreased between 60 and 480 minutes [11]. In the present study, these delayed adverse effects could not be observed in the short time before general anesthesia was scheduled. Differences between experimental protocols may explain the varied results.

Dexmedetomidine used intravenously during or at the end of general anesthesia to decrease narcotic consumption or inhibit the stress response of extubation does not result in delayed recovery [32]. Because much less dexmedetomidine was used in our study than that used in general anesthesia, delayed recovery did not occur following general anesthesia in either group. Because propofol and remifentanil were infused for general anesthesia maintenance, the sedative effects of dexmedetomidine were not detected. High doses of dexmedetomidine did not damage the axon or myelin in animal experiments, and reportedly reduce the acute perineural inflammation caused by bupivacaine [17] and neuroapoptosis induced by volatile anesthetics [33]. We detected no persistent groin pain, painful paresthesia, or neuropathy in the present study, which is consistent with these previous findings.

This study had some limitations. First, the method of ultrasound-guided regional anesthesia has been accepted as the “gold standard” and enables a reduction in local anesthetic use [34,35]. We used a nerve stimulator, rather than ultrasound, for ONB. Further studies are needed to evaluate the combination of ultrasound-guided block and dexmedetomidine. Second, the dose of dexmedetomidine used for the peripheral nerve block was empirically chosen, based on previous studies [10,11,21]. Brummett discovered that dexmedetomidine prolonged sciatic nerve blocks in a dose-dependent manner [18]. A dose-response study should be performed to evaluate the effect of different doses and determine the optimal concentration of dexmedetomidine.

In conclusion, the addition of 1 μg/kg dexmedetomidine to lidocaine for inguinal approach ONB reduced the median effective concentration of lidocaine. Adverse effects such as bradycardia, delayed recovery, and peripheral nerve toxicity did not occur. Further studies are needed to evaluate the combination of ultrasound-guided block and dexmedetomidine, and to determine the optimal dosage of dexmedetomidine for peripheral nerve blocks. Dexmedetomidine may also be useful for other types of surgery, and should be studied in a larger study population and in different nerve blocks.

## Supporting Information

S1 CONSORT Checklist Consort 2010 Checklist.doc. (DOC)

S1 File Letter from ethical committee for approval. (PDF)

S2 File Original data collected from research, presented in table format. (XLS)

S1 Protocol Original trial protocol.doc. (DOC)
