# Supplementary material for: Perineural Dexmedetomidine as an Adjuvant Reduces the Median Effective Concentration of Lidocaine for Obturator Nerve Blocking: A Double-Blinded Randomized Controlled Trial
Source: PLoS One. 2016 Jun 24;11(6):e0158226. doi: 10.1371/journal.pone.0158226 (PMC4920423; doi:10.1371/journal.pone.0158226)
Supplement: S1 File — (PDF) [file pone.0158226.s002.pdf]

ZYY-IRB-SOP-016(F)-002-02

伦理审查批件

批件号	IRB2013-037-01		
项目名称	闭孔神经阻滞穿刺入路及用药剂量的研究<br>——前瞻性、随机、盲法研究		
项目来源	自筹		
研究单位	天津医科大学总医院麻醉科		
主要研究者	王国林、卢悦淳		
审查类别	初始审查	审查方式	会议审查
审查日期	2013年5月30日	审查地点	天津医科大学总医院科学会议厅
审查委员	邵宗鸿、薛凤霞、于士柱、张建宁、章志翔、才华、古津贤、张庆瑜、程焱、林梅		
批准文件	临床研究方案、知情同意书		

审查意见：

根据卫生部《涉及人的生物医学研究伦理审查办法(试行(2007))》、SFDA《药物临床试验质量管理规范(2003)》、《医疗器械临床试验规定(2004)》、WMA《赫尔辛基宣言》和CIOMS《人体生物医学研究国际道德指南》的伦理原则，经本伦理委员会审查，同意按所批准的临床研究方案、知情同意书、招募材料开展本项研究。

请遵循GCP原则、遵循伦理委员会批准的方案开展临床研究，保护受试者的健康与权利。

研究开始前，请申请人完成临床试验注册。

研究过程中若变更主要研究者，对临床研究方案、知情同意书、招募材料等的任何修改，请申请人提交修正案审查申请。

发生严重不良事件，请申请人及时提交严重不良事件报告。请按照伦理委员会规定的年度/定期跟踪审查频率，申请人在截止日期前1个月提交研究进展报告；申办者应当向组长单位伦理委员会提交各中心研究进展的汇总报告；当出现任何可能显著影响试验进行或增加受试者危险的情况时，请申请人及时向伦理委员会提交书面报告。

研究纳入了不符合纳入标准或符合排除标准的受试者，符合中止试验规定而未让受试者退出研究，给予错误治疗或剂量，给予方案禁止的合并用药等没有遵从方案开展研究的情况；或可能对受试者的权益、健康以及研究的科学性造成不良影响等违背GCP原则的情况，请申办者/监查员/研究者提交违背方案报告。

申请人暂停或提前终止临床研究，请及时提交暂停/终止研究报告。

完成临床研究，请申请人提交结题报告。

年度/定期跟踪审查频率	12个月，请于2014年5月29日前一个月提交研究进展报告
有效期	12个月
联系人与联系电话	金冬来　022-60361044
主任委员签字	[illegible]
伦理委员会	天津医科大学总医院医学伦理委员会（盖章）
日期	2013 年 5 月 30 日

Translation:

ZYY-IRB-SOP-016(F)-002-02

Ethical Review for Medical Research

Permission Number	IRB2013-037-01		
Research Title	Perineural Dexmedetomidine as An Adjuvant to Lidocaine for Obturator Nerve Block		
Funding	No Funding		
Institution	Department of Anesthesiology, 2nd Hospital of Tianjin Medical University		
Main Researchers	Wang Guolin, Lu Yuechun		
Review Stage	Preliminary Review	Review Type	Meeting
Review Date	2013-May-30th	Review Site	Tianjin Medical University General Hospital Main Meeting Room
Review Committee	Shao Zonghong, Xue Fengxia, Yu Shizhu ,Zhang Jianning, Zhang Zhixiang, Cai Hua, Gu Jinxian, Zhang Qingyu, Cheng Yan, Lin Hai		
Document for Approvement	Original Trial Protocol		
Review Comments			
According to "Approaches to the Ethical Review of Biomedical Research Involving Human(2007)", "Standard for Quality Management of Drug Clinical Trials(2003)", "Helsinki Declaration" and "International Ethical Guidelines for Biomedical Research in Human Body", after a review of the ethics committee, we agrees to carry out clinical research in accordance with the approved clinical research program.<br>Please follow the "Regulation of Drug Clinical Trial Management" principle, insist on the research plan approved by the ethics committee and protect the health and rights of the subjects. Before your research, please perform trial registration in ClinicalTrails.<br>If the main researchers, subjects or trail protocol has to be changed during the experiment, please submit application for amendment.<br>If serious adverse events occur, please submit a report of serious adverse events in a timely manner. If there's any possible significant effects that could affect the experimental performance or significantly harm the subjects, please submit a written report to the ethics committee immediately.<br>If the research is not insist on the trail protocol approved, or if it is possible to have			

a negative impact on the interests, health of the subjects or scientific research, please submit a fault report.<br>If the study is suspended/early terminated, please submit relevant report for suspension/early termination.	
Time for Check	12months / Please submit research progress before 2014-May-29th
Research Period	12 Months
Contacts & Tel	Jin Donglai +86-22-60361044
Chairman	Shao Zonghong
Ethics committee	Committee on the Ethics of Experiments of Tianjin Medical University General Hospital, Tianjin, China
Date	2013-May-30th
