# Supplementary material for: Perineural Dexmedetomidine as an Adjuvant Reduces the Median Effective Concentration of Lidocaine for Obturator Nerve Blocking: A Double-Blinded Randomized Controlled Trial
Source: PLoS One. 2016 Jun 24;11(6):e0158226. doi: 10.1371/journal.pone.0158226 (PMC4920423; doi:10.1371/journal.pone.0158226)
Supplement: S1 Protocol — (DOC) [file pone.0158226.s004.doc]

**CLINICAL TRIAL PROTOCOL**

**FOR TIANJIN MEDICAL UNIVERSITY**

**1 Study title**

Perineural Dexmedetomidine as An Adjuvant of Lidocaine for Obturator Nerve Block

**2 Investigators**

# Guolin Wang, Department of Anesthesiology, Tianjin Medical University General Hospital, Tianjin ,China.

# Yuechun Lu, Department of Anesthesiology, 2nd Hospital of Tianjin Medical University, Tianjin, China.

# Jian Sun, Department of Anesthesiology, 2nd Hospital of Tianjin Medical University, Tianjin, China.

# Xinqi Zhuang, Department of Anesthesiology, 2nd Hospital of Tianjin Medical University, Tianjin, China.

# Guoyi Lv, Department of Anesthesiology, 2nd Hospital of Tianjin Medical University, Tianjin, China.

# Yize Li, Department of Anesthesiology, Tianjin Medical University General Hospital, Tianjin ,China.

# Haiyun Wang, Department of Anesthesiology, Tianjin Medical University General Hospital, Tianjin ,China.

**3 Background**

# Transurethral resection of bladder tumors (TUR-BT) involving the lateral wall performed with the resectoscope entails a risk for stimulation of the obturator nerve [1]. The nerve passes in close proximity to the lateral bladder wall and its stimulation results in a sudden, violent thigh adductor spasm. The complications of thigh adductor spasm such as bladder perforation, dissemination of cancer cells [2], vascular injury [3], are disastrous and even life-threatening.

# Different strategies are adopted to avoid these complications during surgery such as use of general anesthesia with muscle relaxants [4] or the use of obturator nerve blockade (ONB) [5,6]. Selective ONB along with intervertebral anesthesia is effective to prevent thigh adductor spasm [5,6] as well as avoid the complications of general anesthesia in old patients. As local anesthetic is needed in both intervertebral block and ONB, the quantity of local anesthetic should be considered to avoid intoxication, especially for patients who need ONB in both sides. The less, the better.

# Recently, dexmedetomidine has been found to be an effective adjuvant to local anesthetics in peripheral nerve blocks. On one hand, most researches focused on upper limbs regional techniques [7-10], the report of its use in lower limbs are still limited [11], and its use in obturator plexus block has not been reported. On the other hand, the addition of dexmedetomidine to nerve plexus blocks resulted in a shorter onset time [7-9], an extended duration [7-9,11], and a longer postoperative painless period [7-9,11]. Whether dexmedetomidine could decrease the quantity of local anesthetic is still uncertain.

### 4 Trial Objective

# The aim of the present study was to investigated the effects of dexmedetomidine as an adjuvant on the medium effective concentration(EC50) of lidocaine for obturator nerve block during transurethral resection of bladder tumor.

**5 Study Population**

60 patients with ASA physical status I and II , who were scheduled to undergo elective TUR-BT on lateral in 2nd Hospital of Tianjin Medical University are enrolled in the study.

## 6 Trial Design

**6.1 Grouping and Trail Protocol**

The study is designed as a prospective, double blinded, single center randomized controlled trial. The total number of patients enrolled into this trial will be 60.

Using a computer-generated sequence of numbers, patients were randomly assigned to receive either lidocaine group (group C, 30 patients) or dexmedetomidine group (group D,30 patients) . In group C, only lidocaine was used in ONB while in group D, a the mixture of lidocaine and 1μg/ kg dexmetomedine was used.

The Dixon "up-and-down" sequential allocation method [12] was used to determine the EC50 of lidocaine, running the two groups in parallel. The concentration of lidocaine for the second and subsequent patients in each group were dictated by the response of the previous patient in the group, such that an effective block led to a decrease by a factor of 1.2 in concentration of the next patient, an ineffective block led to an increase by a factor of 1.2, while a technical failure led to the exclusion from analysis of the patient with replacement by the next recruited patient using the same concentration. The initial concentration of lidocaine was 1.5%, and 1:200,000epinephrine was added to lidocaine in all patients.

**6.2 Obturator Nerve Block**

Patients were give atropin at 0.5mg intramuscularly before the block. After insertion of a 20-gauge iv cannula in the arm, a 5 mL/kg/h infusion of lactated ringer's solution was started. After standard anesthesia monitoring, baseline measurements of electrocardiogram (ECG), noninvasive arterial blood pressure (NBP), peripheral oxygen saturation (SpO2), and respiratory rate (RR) were recorded before the block was performed. ONB was performed on the right or/and left sides according to the approach assignment for the side. All ONB were performed by a single experienced investigator not involved in further peri-operative care of those patients.

The point for puncture was identified and ONB was performed as previously described [13,14]. The patient was placed in a supine position, then the legs was slightly abducted. Then draw a line between the inner border of the adductor longus tendon and the most obvious point of the femoral arterial pulse along the inguinal crease on the skin. The midpoint was marked for puncture. A 21 gauge 100 mm needle connected with a peripheral nerve stimulator was inserted in a 30º cephalad direction to the skin. Initially, a current of 2mA at a frequency of 2Hz was set. Once the needle was in contact with the anterior branch of obturator nerve, gracilis or adductor longus contraction was elicited. The current was gradually turned down until muscle contractions occured at 0.4-0.5mA. After aspiration was negative, 5ml lidocaine with or without dexmedetomidine was injected. Then inserted the needle more deeply at 5º lateral direction until the adductor magnus were elicited which means the posterior branch was detected. The rest operation is the same as anterior branch.

**6.3 Block Evaluation.**

**6.3.1 Leg lift scale**

0= patient can lift his leg without abduction and adduct his leg without effort. 1= patient lifts his leg with slight abduction and adduct leg with small effort. 2= patient lifts his leg with obvious abduction and adduct his leg with much effort. 3= patient lifts his leg with much effort and obvious abduction and/or cannot adduct his leg at all.

**6.3.2 Measurement of the abductor muscle strength**

Put a mercury sphygmomanometer previously inflated to 40mmHg between the patient’s knees, the patient was asked to stretch his leg and squeeze the blood pressure cuff with maximum effort by adduct the leg while the observer fix his contralateral leg toward the midline to ensure the pressure generated on the cuff can reflex the strength of only the blocked leg. The maximal sustained pressure read on the mercury sphygmomanometer was regarded as the adductor muscle strength of the blocked leg[15].

**6.3.3 Evaluation of leg movement during operation**

0=leg abducted violently during resection of the bladder tumor and the operation was difficult to continue. 1=leg abducted slightly during resection of the bladder tumor and the operation can continue with care. 2=leg did not move at all during resection of the bladder tumor.

**6.3.4 The identification of a successful ONB**

The identification of a successful ONB should satisfy all the following standards within 10 minutes after ONB: leg lift scale got “2” or “3”, adductor muscle strength decreased ≥50% and leg movement evaluation got “2”. As to the failure case, intravenous cisatrocuronium was offered at 10mg or more if necessary before operation.

**6.4 Anaesthetic Management**

As compensation for nerve block and facilitation of postoperative evaluation, general anesthesia was scheduled after block evaluation. Induction was performed and maintained by propofol and remifentanyl intravenously. Ventilation was sustained with LMA. Perioperative monitoring included pulse oximetry (SpO2), electrocardiogram (ECG), non invasive arterial pressure (NBP), bispectral index (BIS), temperature and partial pressure of carbon dioxide in endexpiratory gas(PETCO2). The liquid for bladder flushing was heated to 30℃ in order to maintain formal body temperature. If the heart rate declined less than 40 bpm or decreased more than 30% of the basic value, it was recorded as bradycardiac and intravenous atropine was given.

LMA was pulled out after the recovery of patient’s spontaneous breathing, consciousness and physical reflex postoperatively. Delayed recovery was defined as LMA could not be pulled out 30 minutes after operation. Time interval between the completion ONB and TUR-BT was recorded. In PACU, adductor muscle strength was evaluated again after performing successful block. Within 24 hrs after the study, patient underwent a examination regarding adverse events such as persistent groin pain, painful paresthesia, and neuropathy.

**6.5 Measures to avoid /minimize bias**

**6.5.1 Randomization**

Patients will be randomized using a computer generated randomization procedure. Patients will be randomly assigned to receive either “lidocaine group” or “dexmedetomidine group”.

**6.5.2 Blinding**

All patients do not know the group they allocated to. And all obturator nerve block were performed by a single experienced investigator not involved in further peri-operative care of those patients. Thus, he is unable to select the drug during the operation, and do not know to which group the specific patient was allocated to.

**6.6 Duration of study participation**

The participation duration per patient will be 24 hrs after the study, patient underwent a examination regarding adverse events such as persistent groin pain, painful paresthesia, and neuropathy. In case of persistent adverse events and/or intercurrent diseases, appropriate medical measures have to be taken; in such circumstances the patients have to be closely monitored until the problems are solved.

**6.7 Stopping rules or discontinuation**

A patient may be withdrawn from the study for the following reasons:

- Withdrawal of consent
- Not cooperative, non compliance
- Obturator nerve block could not be performed on the patient for any reason.
- Any reason which, in the eyes of the investigator, does not justify continuation of the study
- Serious adverse events / intra-operative events : myocardial infarction, admission to intensive care after operation, peroperative blood loss of > 50% of circulating volume (usually set at 70 ml/kg BW for an adult)

**7 Selection and Withdrawal of subjects**

Prior to possible recruitment, all patients will be thoroughly examined. They will undergo detailed physical examination (screening) including the following:

- Medical History
- Age, weight, height, ethnic origin, sex.
- Previous anaesthesia
- Concomitant diseases
- Concomitant medication
- ECG (12-lead)
- Body weight
- Non invasively derived Blood pressure.
- Laboratory tests (including level of Haemoglobin, and plasmalevels of sodium, potassium, urea and creatinine,)

**7.1 Subject inclusion criteria**

The patients must comply with the following criteria in order to be eligible to participate in this clinical study:

- Age range ≥ 18 years
- ASA classification I – II
- Patients who will undergo elective TUR-BT on lateral
- Written informed consent

All inclusion criteria must be met; otherwise the patient cannot be enrolled into the study.

**7.2 Subject exclusion criteria**

During the study period, it must be demonstrated that the following criteria do not apply:

- Age range: < 18 years
- ASA classification III, IV or V
- Emergency operations
- Pregnancy
- Breast feeding period
- Informed consent missing
- Alcohol abuse (more than 35 units a week)
- Drug abuse (opiates, cocaine)
- abnormal coagulation function
- neuromuscular disease
- diabetes mellitus
- SaO_2_ < 90% (room atmosphere) SpO_2_< 8 kPa
- Legal incapacity
- Refusal to undergo obturator nerve block
- Dialysis or fluid restriction based on renal failure
- a known allergy to local anesthetics or other medications used in this study
- Any clinical condition which does not justify study participation in the investigator’s opinion .

If one or more of these criteria apply, the patient will not be enrolled into this study.

**7.3 Type and timing of the data to be collected for withdrawn subjects**

In case of withdrawn subjects the reasons for withdrawal will be accurately documented in the article.

**8 Adverse events**

**8.1 Definition of Adverse Events/Effects**

Adverse events are defined as symptoms or events reported spontaneously by the patient or detected by the investigator and occur in a patient. The necessity to document this information is independent of whether this event is related to the administration of a ““lidocaine” group or “dexmedetomidine” group.

**8.2 Severity**

All adverse events and/or symptoms have to be rated according to severity, seriousness, and possible relation to the study.

The severity of each adverse event has to be classified as *mild, moderate, severe* or *extreme,* according to the following guidelines:

*Mild:* No influence on the daily activities of the patients, no symptomatic treatment necessary.

*Moderate:* Normally associated with a certain inconvenience or concern to the patients, may influence daily activities, optional use of simple therapeutic measures.

*Severe:* Associated with a considerable inconvenience or concern to the patients and necessitates in general medical treatment or other appropriate therapeutic measures.

*Extreme*: Interrupts the daily activities of the patients and necessitates medical treatment or other appropriate therapeutic measures.

**8.3 Recording of Adverse Events/Effects**

Any adverse event must be recorded. In this section the following data will be collected:

- Kind of adverse event
- Onset
- Duration
- Severity
- Course
- Relation to concomitant diseases/treatments
- Relation to the study drugs

Special attention has to be paid to this section, as the evaluation of potential risks is of major importance in terms of the benefits gained by the patients. Besides, all medical interventions have to be specified in this sheet.

**9 Time Schedules**

After approval by the ethics committee patient enrollment should start by the first quarter of 2014.

**10 Statistics**

**10.1 Statistical analysis**

Besides the goal of estimating the EC50 of lidocaine from the up-and-down study, it is also necessary to specify the precision of the target dose with a 95% confidence interval (CI). Drug effect increases with increasing concentration, but biologic and experimental variability may produce unexpected ups and downs in the observed response rate as concentration increases. Thus, the adjusted response rates were calculated by the pooled-adjacent-violators algorithm (PAVA) as adjusted response rates. The isotonic estimator of target dose with isotonic regression provides a smaller bias, mean square error, greater precision (tighter CI), and requires no symmetry assumption [16]. The EC50 of lidocaine and CIs were calculated from R version 3.0.1(R foundation for Statistical Computing, Vienna, Austria). Comparison of EC50 between groups used the method of isotonic regression.

For all patients, sex constitution, age, body mass index(BMI), ASA grade, time interval between the completion of ONB and TUR-BT, adductor muscle strength evaluation after ONB and in PACU, and the occurrence of bradycardiac, delayed recovery, groin pain, painful paresthesia, and neuropathy, were recorded. The data above are presented as mean±standard deviation, or count as appropriate. Statistical comparisons of continuous variables were performed using Student’s t-test and categorical variables were analysed using χ2 test with SPSS software, version 19.0 (SPSS, Inc., Chicago, Illinois, USA). Significance was defined at P<0.05.

**10.2 Sample size**

The Dixon "up-and-down" sequential allocation method [12] was used to determine the EC50 of lidocaine. In this method, the stopping rule required at least 6 failure/success pairs. As a result, 30 patients were enrolled in each group to confirm at least 7 crossovers.

**11 Records**

**11.1 Entries**

All data entries have to be made directly in the case record for each patient. The investigator will be responsible for ensuring that all the questions on the record are answered fully. If certain data are not available, not done or not applicable the investigator will enter: “N.Av.”, “N.D.”, “N.Ap”, respectively, in the appropriate spaces. The 24-hour clock will be used for all time entries. (00:00 – 23:59)

**11.2 Errors**

The case record will be reviewed and evaluated for completeness and forms with errors or omissions will be returned to the investigator for correction. Changes or additions to the data must be made in the following manner: the original entry will be crossed out with a single line drawn through the error (not erased or whited out) so as to leave the original entry still legible. The correction should be entered in ink, initialled and dated by the person making the correction.

**11.3 Maintenance of records**

The investigator will keep a record of the full names and addresses of the patients and a copy of the signed informed consent form, which must be able to uniquely identify a patient, and the corresponding study number. These records are to be retained for a period of 15 years following the completion of the trial.

**12 Ethics**

**12.1 Informed consent**

A physician will inform the patients or patients relative about the nature, relevance and consequences of the study and they have to give their written informed consent to participate in this study. Additionally they will receive written study information.

**12.2 Ethics committee**

The study protocol and the appropriate documents have been submitted and approved by the Ethics Committee.

**12.3 Declaration of Helsinki**

The investigators pledge to fully respect and follow the Declaration of Helsinki in the current version of Somerset West, SA (1996)^27^

**13 References**

1. Hradec E, Soukup F, Novák J, Bures E. Preventing damage of the bladder wall during transurethral surgery. Int Urol Nephrol 1983;15(2):149-53.PMID: 6629690

2. Tekgül ZT, Divrik RT, Turan M, Konyalioğlu E, Şimşek E, Gönüllü M. Impact of obturator nerve block on the short-term recurrence of superficial bladder tumors on the lateral wall. Urol J. 2014 Mar;11(1):1248-52.PMID: 24595932

3. Akata T, Murakami J, Yoshinaga. A Life-threatening haemorrhage following obturator artery injury during transurethral bladder surgery: a sequel of an unsuccessful obturator nerve block. Acta Anaesthesiol Scand. 1999 Aug;43(7):784-8.PMID: 10456822

4. Berglund RK, Herr HW. Surgery for bladder cancer. In: Walsh PC, editor. Campbell’s Urology. 8th ed. Philadelphia: WB Saunders;2002. pp. 2375-85.

5. Deliveliotis C, Alexopoulou K, Picramenos D, Econornacos G, Goulandris N, Kostakopoulos A. The contribution of the obturator nerve block in the transurethral resection of bladder tumors. Acta Urol Belg. 1995 Sep;63(3):51-4.PMID: 7484523

6. Tatlisen A, Sofikerim M. Obturator nerve block and transurethral surgery for bladder cancer. Minerva Minerva Urol Nefrol. 2007 Jun;59(2):137-41.PMID: 17571049

7. Esmaoglu A, Yegenoglu F, Akin A, Turk CY. Dexmedetomidine added to levobupivacaine prolongs axillary brachial plexus block. Anesth Analg. 2010 Dec;111(6):1548-51. doi: 10.1213/ANE.0b013e3181fa3095 PMID: 20889939.

8. Marhofer D, Kettner SC, Marhofer P, Pils S, Weber M, Zeitlinger M. Dexmedetomidine as an adjuvant to ropivacaine prolongs peripheral nerve block: a volunteer study. Br J Anaesth. 2013 Mar;110(3):438-42. doi: 10.1093/bja/aes400 PMID: 23161360.

9. Agarwal S, Aggarwal R, Gupta P. Dexmedetomidine prolongs the effect of bupivacaine in supraclavicular brachial plexus block. J Anaesthesiol Clin Pharmacol. 2014 Jan;30(1):36-40. doi: 10.4103/0970-9185.125701 PMID: 24574591.

10. Song JH, Shim HY, Lee TJ, Jung JK, Cha YD, Lee DI, et al. Comparison of dexmedetomidine and epinephrine as an adjuvant to 1% mepivacaine in brachial plexus block. Korean J Anesthesiol. 2014 Apr;66(4):283-9. doi: 10.4097/kjae.2014.66.4.283 PMID: 24851163.

11. Rancourt MP, Albert NT, Cote M, Letourneau DR, Bernard PM. Posterior tibial nerve sensory blockade duration prolonged by adding dexmedetomidine to ropivacaine.Anesth Analg. 2012 Oct;115(4):958-62 PMID: 22826530.

12. Dixon WJ. Staircase bioassay: the up-and-down method. Neurosci Biobehav Rev. 1991;15(1):47-50.

13. Jo YY, Choi E, Kil HK. Comparison of the success rate of inguinal approach with classical pubic approach for obturator nerve block in patients undergoing TURB. Korean J Anesthesiol. 2011 Aug;61(2):143-7. doi: 10.4097/kjae.2011.61.2.143 PMID: 21927685.

14. Moningi S, Durga P, Ramachandran G, Murthy PV, Chilumala RR. Comparison of inguinal versus classic approach for obturator nerve block in patients undergoing transurethral resection of bladder tumors under spinal anesthesia. J Anaesthesiol Clin Pharmacol. 2014 Jan;30(1):41-5. doi: 10.4103/0970-9185.125702 PMID: 24574592.

15. Lang SA, Yip RW, Chang PC, Gerard MA. The femoral 3-in-1 block revisited. J Clin Anesth. 1993 Jul-Aug;5(4):292-6. PMID: 8373606.

16. Pace NL, Stylianou MP. Advances in and limitations of up-and-down methodology: a precis of clinical use, study design, and dose estimation in anesthesia research. Anesthesiology. 2007 Jul;107(1):144-52. PMID: 17585226.
